# Gatekeepers or Intermediaries? The Role of Clinicians in Commercial Genomic Testing

**DOI:** 10.1371/journal.pone.0108484

**Published:** 2014-09-26

**Authors:** Michelle L. McGowan, Jennifer R. Fishman, Richard A. Settersten, Marcie A. Lambrix, Eric T. Juengst

**Affiliations:** 1 Department of Bioethics, Case Western Reserve University School of Medicine, Cleveland, Ohio, United States of America; 2 Biomedical Ethics Unit, McGill University Faculty of Medicine, Montreal, Quebec, Canada; 3 College of Public Health and Human Sciences, Oregon State University, Corvallis, Oregon, United States of America; 4 Department of Bioethics, Case Western Reserve University School of Medicine, Cleveland, Ohio, United States of America; 5 Center for Bioethics, Department of Social Medicine, University of North Carolina – Chapel Hill, Chapel Hill, North Carolina, United States of America; Kilifi, Kenya

## Abstract

**Background:**

Many commentators on “direct-to-consumer” genetic risk information have raised concerns that giving results to individuals with insufficient knowledge and training in genomics may harm consumers, the health care system, and society. In response, several commercial laboratories offering genomic risk profiling have shifted to more traditional “direct-to-provider” (DTP) marketing strategies, repositioning clinicians as the intended recipients of advertising of laboratory services and as gatekeepers to personal genomic information. Increasing popularity of next generation sequencing puts a premium on ensuring that those who are charged with interpreting, translating, communicating and managing commercial genomic risk information are appropriately equipped for the job. To shed light on their gatekeeping role, we conducted a study to assess how and why early clinical users of genomic risk assessment incorporate these tools in their clinical practices and how they interpret genomic information for their patients.

**Methods and Findings:**

We conducted qualitative in-depth interviews with 18 clinicians providing genomic risk assessment services to their patients in partnership with DNA Direct and Navigenics. Our findings suggest that clinicians learned most of what they knew about genomics directly from the commercial laboratories. Clinicians rely on the expertise of the commercial laboratories without the ability to critically evaluate the knowledge or assess risks.

**Conclusions:**

DTP service delivery model cannot guarantee that providers will have adequate expertise or sound clinical judgment. Even if clinicians want greater genomic knowledge, the current market structure is unlikely to build the independent substantive expertise of clinicians, but rather promote its continued outsourcing. Because commercial laboratories have the most “skin in the game” financially, genetics professionals and policymakers should scrutinize the scientific validity and clinical soundness of the process by which these laboratories interpret their findings to assess whether self-interested commercial sources are the most appropriate entities for gate-keeping genomic interpretation.

## Introduction

As the cost of genomic analysis has plummeted over the last decade, genomic risk profiling has become increasingly available in consumer and clinical settings. In 2007, commercial laboratories began offering consumers products to assess their inherited risks for a variety of complex diseases and traits by analyzing single nucleotide polymorphisms (SNPs) across the genome [1]. These companies ignited a firestorm by making their tests available directly to consumers (DTC) for purchase via the internet, thereby disrupting the typical pathway of development and dissemination for genetic risk information [2]. Critics and regulatory agencies raised concerns that offering genomic test results DTC with insufficient knowledge and training in genomics may pose harms to consumers, the health care system, and society [3]–[5], as these tests are non-diagnostic and rarely indicate a clear clinical course of action [2]. Advocates of DTC genomic risk profiling touted consumer genomics’ potential to improve the practice of medicine by empowering patients to independently obtain personal risk information and then inform their own healthcare through collaboration with their physicians [6]–[8]. Yet, research suggests that general practitioners and genetics specialists alike feel unprepared to interpret and act on patients’ DTC genomic test results in their clinical practices – whether because they lack knowledge, familiarity, or confidence in genomics or because their skepticism toward commercial testing platforms hinders their comfort and assuredness in counseling patients on the basis of these results [9]–[16]. As a result, critics argue that “knowledgable genetics professionals” – employed independently of commercial laboratories – should be gatekeepers to commercial genomic testing because ordering, interpreting and returning genomic information “requires competent (deliberative, evidence-based, rigorous, and accountable) clinical judgment” [3], [17].

In response to regulatory scrutiny, the DTC marketing approach has faltered [18], [19]. Several commercial laboratories have retreated from selling tests DTC and adopted a more traditional “direct-to-provider” (DTP) marketing model [1], which repositions physicians – most often general practitioners – as the intended recipients of advertising of laboratory services and as gatekeepers to personal genomic information [4], [20]. This shift is generally attributed to the declining costs of genome sequencing, the slow uptake of DTC services in the population, and pressures exerted from the Food and Drug Administration (FDA) and medical professional societies on the genomics industry to comply with laboratory and medical device standards [1], [21]. Some laboratories have even taken the stance that if genomic analysis is to have any impact on how clinicians practice medicine, it will be through direct relationships with companies rather than through patients who provide profiles they have personally acquired [21]. The retreat from the DTC model has been applauded by those who consider professional clinical judgment indispensable in the interpretation of results provided by commercial laboratories [3], [22].

As enthusiasm for clinical applications of next generation sequencing (NGS) grows [23], so does the challenge of understanding, translating and managing genomic risk assessment. Clinical sequencing will require not only the ability to interpret patient data and answer patient questions about specific genomic risks, but also to address the “incidentalome” of other information of variable significance that comes with it [24]. In addition, professional genetics societies are generating growing lists of genetic mutations that are deemed predictive enough of preventable risks to warrant opportunistic sequencing by laboratories whenever clinical sequencing is ordered [25]. Meanwhile, even as the feasibility of clinical sequencing is being studied by the National Institutes of Health, commercial genomics laboratories such as Ambry Genetics, DNA DTC, Gene Dx, and Illumina are now marketing both targeted and whole exome sequencing DTP. These companies are also promising to convey opportunistic “secondary” sequencing results alongside the requested information for physicians to convey to patients as they see fit [26]–[29].

These factors put a premium on making sure that those who are charged with interpreting, translating, communicating and managing commercial genomic risk results are appropriately equipped for the job. Do primary care physicians and other health professionals who order commercial genomic tests and receive results have the skills and knowledge necessary to make sound clinical judgments? If not, where will they obtain that knowledge? Research suggests that primary care physicians who feel well-informed about genetic testing are receptive to incorporating genomic risk profiling into their practices [30], but little is known about their knowledge of genomics and the factors that contribute to their decisions to offer genomic testing to patients. To shed light on these issues, we turn to data from a study of early clinical users of genomic risk assessment. The perspectives of early-adopting clinicians are valuable because early adopters are typically defined by their willingness to promote and adapt technology to suit their own use and by their contributions to shaping its future use [31]. Given the likelihood that genomic risk assessment tools will be further integrated into clinical care in the future, it is particularly important to understand the ideological beliefs, knowledge, and practices of early adopting clinicians.

## Methods

The study was designed to assess how early clinical users of genomic risk assessment understood genomics and used their knowledge of genomics to inform clinical decisions. The data presented here was collected as a component of a larger study investigating how the goals, benefits, challenges, and consequences of translational genomic research (TGR) and personalized genomic medicine (PGM) are interpreted and anticipated by its proponents. The broader study involved interviews with leaders in key stakeholder groups [32], such as research funders, scientists, journal editors, clinicians, educators, and entrepreneurs. Data presented here are focused on interviews conducted with clinicians implementing PGM in their practices. This group represented a distinct vantage point with respect to the clinical translation of genomic information.

We employed a purposive sampling strategy to identify early clinical adopters of DTP genomic risk assessment. We first reviewed the member organizations under “Consumer Genetic Testing Services” on the Personalized Medicine Coalition website and visited each organization’s website to determine whether consumer genetic testing services were accompanied by information or services provided by partnering clinicians. Of these, two commercial genetic testing laboratories indicated that they offered genomic services to patients through a certified health care professional.

Hence, our sample was comprised of clinician partners of these two commercial genetic testing laboratories: DNA Direct, a commercial laboratory that provides decision support tools and genetic counseling support to clinicians to help them incorporate personalized genomic medicine into their clinical practices [33]; and Navigenics, a highly influential but now defunct commercial laboratory that established collaborative partnerships with concierge physicians to offer SNP-based genomic risk assessment as a service to their patients [34].

Participants were recruited from 17 clinical sites (16 in the U.S. and one in Canada) listed as partners on the DNA Direct and Navigenics websites in 2011 Of the 91 clinicians affiliated with these sites, 18 clinicians agreed to participate, 37 declined, 23 were unresponsive, and 13 were determined to be ineligible either because the recruitment letter was returned as undeliverable or because the clinician had left the practice. While the response rate and sample size may be considered limitations of the study, the response rate is consistent with other studies involving physician recruitment for qualitative interviews [35] and provided sufficient data to achieve theoretical saturation [36]. We cannot claim that the views of respondents are representative of the pool of clinicians from which they were recruited, but they are demographically similar to non-respondents in terms of educational attainment and clinical practice affiliation. The timeframe in which the study was conducted may also be seen as a limitation of the study as the commercial genomic testing approach of partnering with clinicians has expanded since the data was collected in 2011. However, these data provide a novel set of perspectives of early clinical users of genomic risk assessment that can be instructive as commercial genomic testing becomes further integrated into clinical care.

This study was approved by the Case Western Reserve University IRB (approval number 20100801). Participants provided oral consent to participate in a phone interview with one of three study research associates, which was documented in a password-protected database. Because interviews were conducted by phone and for the ease of to obtaining consent in real time, the IRB approved our request to seek oral consent over written consent for this study. Once the interviewer turned on the audio recording device, he or she asked the interviewee whether they would provide oral consent to participate in the study. The interviewee’s response was then captured on the interview recording.

Interviews probed clinician perspectives on and experiences with partnering with commercial genomics laboratories and offering genomic risk profiling to their patients (see Appendix S1 for the interview guide). Interviews were conducted by phone, digitally-recorded and transcribed for thematic analysis. In an iterative process combining inductive and deductive methods, the research team used a subset of transcripts to generate a coding scheme [36]. To enhance inter-coder reliability, the research team developed a codebook of definitions and examples for each code and rules for applying codes to the transcripts [37], [38], and coded a batch of initial interviews together to refine codes and coding rules [39]. Interview transcripts were double-coded by two research assistants and reviewed for reliability using Atlas.ti 6 qualitative analytic software. Areas of disagreement were then reviewed to achieve consensus. The research team drafted summaries of coded data, working across summaries to identify larger themes [39], [40].

## Results

We conducted a total of 18 interviews with clinicians affiliated with 16 clinical sites in the United States and 1 clinical site in Canada. We interviewed 15 primary care physicians, two genetic counselors, and one medical geneticist practicing in community hospitals and private concierge medical practices (where patients pay a retainer fee for individualized, preventive, and wellness-oriented health care from a physician with a low patient load) [41]. The clinicians established relationships with the companies to commission laboratory testing as well as analytic, decision-support, and genetic counseling services to help interpret laboratory-issued genomic test result reports [33], [34]. These clinicians were not employed by the commercial laboratories DNA Direct or Navigenics, and, to our knowledge, did not receive financial incentives for partnering with these commercial laboratories to offer genomic risk assessments to patients.

Most interviewees indicated that they first learned about genomic risk assessment through laboratories’ DTP marketing campaigns, which were aimed toward what one family physician characterized as “physicians that are … looking for more opportunity [sic] for prevention and wellness” (Provider 129). An internist affiliated with a network of concierge medicine providers explained that Navigenics approached his practice:

to see if we would be interested in sending, having our patients genomically tested. So I did a little pilot study to see how we thought genomic testing might work into our practice … of 30 patients that [Navigenics] tested for free whereby either blood or buccal swabs were sent to their lab and then results sent back on those patients … So it was interesting and it was expensive. Well it wasn’t expensive for the patient, ‘cause they were all done for free, but subsequently it’s expensive to do the genetic testing. [Navigenics was] gonna give us a special deal because of our large number of patients throughout the country that might be interested in this, because generally they’re patients that are pretty proactive patients that are really caring about their health and would be willing to be tested for various types of screening that might benefit them. So it was a population that was easily captured for [Navigenics]. (Provider 107)

Clinicians affiliated with concierge practices found the opportunity to offer genomic risk assessment appealing because the laboratories’ values seemed aligned with their own focus on personalized, preventive, and wellness-oriented patient care [42]. Other participants were convinced of the overall value of these services after receiving a discounted or complimentary self-test. One family physician recommended that all physicians considering a partnership with a commercial laboratory undergo genomic risk assessment themselves because “they can start to understand personally how it might actually be beneficial for their patients” (Provider 156). Some clinicians added that incorporating DTP genomic risk assessment into their practices helped give them a competitive edge over other local practices by allowing them to offer the latest screening technologies. To this end, a genetic counselor reported that her patients are “generally expecting … information about risk, about probability that might help them to … be more proactive about their health care. … They’re seeking out these services as one more piece of information that can help them in that area” (Provider 37).

Working with the commercial laboratories was not only seen as a new way to enhance their relationships with patients, but it also allowed these clinicians to tap a knowledge base they did not have or could not otherwise access. As one internist explained, “I knew so many things were happening in genetics, and I knew that some of the tests were hard to order. They were hard to determine which tests to do, and since I had an interest in preventive medicine, the idea that someone had put together a profile of diseases that/where you could do some intervention was great” (Provider 131).

Some clinicians characterized DTP genomic risk assessment as an informational tool that complemented self-reported family history. Others indicated that it had resulted in increased health monitoring to promote patient wellness (e.g., annual CT scans for patients whose results indicated SNPs associated with increased lung cancer risk). Some also characterized the benefit of DTP genomic risk assessment as a way to encourage patients to improve their lifestyles or modify their risk behaviors. For example, one internist ruminated that “it is expensive, but it’s helpful to motivate patients to do lifestyle changes that are difficult, to do different screenings that might be more appropriate for them than if they hadn’t known they were at higher risk for that condition” (Provider 109). A family physician similarly argued, “those [patients] who are getting it done are interested, and [by paying out of pocket] they’ve got skin in the game financially too” (Provider 156).

At the same time, other study participants were skeptical about the value of partnering with commercial laboratories, characterizing genomic risk profiling as an add-on connoisseur service that comes at a high out-of-pocket cost to patients. Citing factors such as prematurity of the science and evidence of the low return on investment, some clinicians, like this internist, argued that “most doctors and most colleagues I have still don’t see the value of Navigenics beyond traditional family history and testing we do, the traditional risk factors” (Provider 138). Even some of the participants who had ordered genomic risk assessments for their own patients, such as the following internist, were skeptical of the value of genomic risk profiling in its present form:

I didn’t feel like it was something strong enough that I could sell… I don’t think it really impacted my practice, and the way I felt about it is that it was just too premature of a science at that point… so I really, I’m like ‘You know what? Let the science work itself out, and then I’ll revisit it in the future.’

In sum, even among clinicians who have entered into relationships with DTP companies and are affiliated with pay-for-service medical practices, there is a striking divergence of views about the medical merits of genomic risk assessment services.

The factor participants cited most commonly as a draw for partnering with commercial laboratories was a knowledge deficit that most providers willingly admitted: insufficient expertise in genomics to independently and critically evaluate and interpret commercial test results, and an inability to help their patients manage genomic risk susceptibility results. Instead, they needed to depend almost exclusively on the laboratories from which they ordered tests. As an internist elaborated:

I have a genetic counselor from Navigenics who’s available to our patients, but really represents an excellent resource for me when I have questions. I have access to be able to ask her questions by email or call her, and so I’m learning on the job. And I’m far from an expert, but I assume I know more than most of my primary care colleagues… A deficiency in moving towards gene-based medicine is that it is difficult to acquire this information, and particularly with busy primary care doctors who just hardly have time to keep up with the usual literature to learn something new. There just isn’t enough time. (Provider 40)

Virtually all of the information study participants used to explain genetic risk susceptibilities to patients came directly from partnering commercial laboratories–through training programs to familiarize clinicians with commercial products and services, pre-test advice on the appropriateness of testing, and/or *ad hoc* counseling by staff genetic counselors to help clinicians interpret test results. As two internists explained:

So basically I had a couple of sessions mostly over the phone, and what [the laboratory] sent me as far as reading material from Navigenics, talking to the genetic counselors– there were two that I spoke with–as well as the PhD geneticist there regarding the technique of the testing and what the SNPs mean and how to really correlate it, you know because it’s really sort of a lifetime genetic, lifetime risk factor you know and how to explain it to the patient. (Provider 107)

The training was a couple of one-hour PowerPoint presentations. Then I have frequent conversations (verbally and email) with my genetic counselor at Navigenics … I’m able to review the results of most patients, but I use her as a resource for questions I have prior to presenting it to the patients. So she’s been a great resource… We [physicians] cannot keep track of all the basic science - that explosion that is occurring logarithmically - but Navigenics does ‘cause I know they update their profiles with each and every major scientific journal. So we rely on them to update their risk analysis based on discovery of new genetic sequences and new risk factors. We rely on them a lot to do that. (Provider 138)

Participants were generally uncritical of and grateful for the training and information that they received from the commercial laboratories. In explaining the value of partnering with commercial laboratories by filling a knowledge gap for ordering clinicians, one family physician explained:

We don’t always have expertise in the field. We don’t know when to initiate a test. We don’t know how to interpret the test. We don’t know how to use the information we got to the patient’s best cause, whereas using the DNA Direct application allows us to make a determination whether or not, as clinicians, we should order this test … then the information comes back to us in a format that we can understand as clinicians. It doesn’t come back in gobbledygook that we don’t understand. The system gives us access to a geneticist so that we’re not left like flapping in the breeze and we’re able to do justice to our community in that we’re able to educate them on where the personalized medicine is important. (Provider 114)

The medical geneticist we interviewed justified this approach by arguing “[I]t’s just a tool. You don’t even need to understand all the medicine or all the genetics or whatever. The same way that we are measuring cholesterol and most people don’t know all the lipid pathways of biochemistry in order to be able to look at the values” (Provider 38). Finally, in articulating why having resources provided by the commercial laboratory was so important to ordering clinicians, a genetic counselor – who presumably would have more specialized training in genomics than a family physician or internist - explained:

I feel like I need to be the expert, even though you know obviously I can’t be an expert in every aspect, and so I feel that DNA Direct is really my fallback, my support system and network you know if I have questions about a case or you know how to follow someone clinically or how to proceed you know with the testing or whatever. Whatever questions I have I can always call DNA Direct, talk with a counselor, get some feedback. Otherwise I think if DNA Direct weren’t here and it weren’t part of my job, I would have a lot of difficulty with trying to stay current and have that support. (Provider 21)

## Discussion

Our study suggests that many early-adopting clinical providers of SNP-based genomic testing were enthusiastic about the potential for these tests to enhance the personalized, preventive and wellness orientations of their clinical practices. However, they largely did not have sufficient knowledge of genomics to independently help their patients manage the genetic risk information that commercial genomic analysis could provide. Instead, clinicians depended on the testing companies themselves to interpret the data in order to counsel their patients. Thus, the retreat to DTP marketing and service delivery model – in which genomic risk assessments are delivered through clinicians to their patients rather than directly to consumers – cannot guarantee the provision of adequate expertise to protect patients or ensure sound clinical judgment on behalf of health care providers. The debate over whether companies or clinicians are better equipped to interpret and counsel the public about the results of commercial genomic risk tests may therefore be misplaced, as the interpretations in either delivery model ultimately comes from the same source. Rather, genetics professionals and policy-makers should scrutinize the scientific validity and clinical soundness of the process by which commercial laboratories interpret their own findings because it is this information that forms the basis of both clinical and public understanding in both DTC and DTP contexts.

Though commercial genomic risk assessment represents only one way that genomics has made inroads into clinical practice, it is often taken for granted that the effective translation of genetics and genomics into primary care requires clinicians’ knowledge and training in applied genetics and genomics [15], [30], [43], [44]. Our results suggest that clinicians do not have the expertise to counsel patients in this regard, even if they have the interest. Moreover, previous research with clinicians who use genomic risk profiling suggests that these clinicians prefer to learn about genomics through continuing medical education, medical literature, formal coursework and seminars [30]. However, the clinicians we interviewed are not gaining knowledge of genomics through these more impartial sources.

The clinicians we interviewed did not express significant concern about the arrangement with commercial laboratories, as it fits with conventional practice regarding the introduction of other medical technologies. Nevertheless, critics have warned that genetic counselors affiliated with commercial laboratories may, more like pharmaceutical representatives than ultrasound technical support staff, be inappropriately or vulnerably positioned to educate physicians about new tests and services [4], [5]. Commercial laboratories have a vested interest in maintaining control over expertise in genomic test development, dissemination, and interpretation, hence may be a biased source of information for clinicians about the benefits and drawbacks of tests [45]. Because clinicians must rely on some other entity for both testing and interpretation, DTP marketing and the educational practices of commercial laboratories demand as much regulatory scrutiny as their DTC initiatives. Neither DTP nor DTC marketing and service delivery models guarantee adequate end user expertise in genomic interpretation. This problem is likely to persist in alternative models too.

Personal genetic information has long been held as exceptional in the clinical and research sphere, on the grounds that genomic risk assessment is more complex, varied and uncertain in meaning than many other tests that clinicians order [46]. Given the potential individual and familial implications of knowledge of one’s heritable risks (whether psychosocial or decisional), Evans and Berg have argued that personal genomic testing should be treated like other “complex medical tests with the power to help, harm, and confuse” [17]. The shifting landscape of genomics and the uncertainty inherent in genomic testing makes results and interpretation less stable than, say, a cholesterol test or a chest film to diagnose pneumonia–particularly because the analytic and clinical validity and clinical utility of commercial genomic tests have yet to be established and are subject to change as new data are generated [22], [47]. McGuire and colleagues have argued that a specialized skill set held by genetics professionals may be required to conduct and return the results of genomic risk assessment [3]. Nevertheless, because primary care providers are likely to receive inquiries about genomic testing from patients, it is important to consider the implications of expanded availability of commercial genomic testing for a wider range of clinicians than traditional genetics specialists.

Primary care physicians may play an important role in integrating genomic risk results by combining information from other diagnostic tools to develop more sensitive treatment plans and advice on how to manage increased disease risks [47]. However, our interviewees – the majority of which were primary care providers – did not convey genomic expertise in ways that demonstrate competent, deliberate, evidence-based clinical judgment in evaluating genomic risks [3]. Rather, the limited genomic expertise of these clinicians more closely resembles “interactional expertise” – the ability to master the rhetorical tools of a specialist domain without practical competence [48]. But clinicians who only possess interactional expertise are not likely to have sufficient competence to assess the validity and utility of commercially-provided risk information. The presumed benefits of having clinicians control genomic information may be further impeded if, as we have suggested in our previous work, patients are more knowledgeable about genomics than their health care providers, which may diminish the confidence patients have in the advice of clinicians regarding genomic risk management [2].

Our findings suggest that clinicians find the DTP marketing model appealing because it offers a new way to enhance relationships with patients and to tap information they would not otherwise have in providing preventive care. But the benefits of the DTP marketing model nonetheless seem weighted toward laboratories. The strategy provides a steady stream of new users to generate profit, and it evades some of the regulatory scrutiny of the DTC model [1] by channeling the information through clinicians as more traditional laboratory tests.

The danger here is that this clinician-laboratory partnership model, in which expertise may be interactional, potentially compromises the integrity of clinical judgment. This is not to discredit the strengths that clinicians may bring to genomic counseling, from professional experience and caution to potential concerns about professional liability. These strengths benefit patients regardless of the physician’s level of genomic expertise, and are applicable any time new technologies are making their way into the clinical encounter. Rather, our goal is to draw attention to the potential for conflicts of interest that emerge when commercial laboratories with a profit motive market their tests to clinicians, and then counsel clinicians on how results should be interpreted and conveyed. Most of our interviewees seemed to lack critical reflection on genomic tests and results, and those that did take a more critical stance typically pointed to the prematurity of integrating genomic science into the clinical encounter rather than with the DTP marketing and service delivery model. It appeared as though participants found it unproblematic to be receiving tests and advise on how to order and interpret test results from the same commercial source. It would be naïve to expect clinicians to develop genomic expertise or critically evaluate all new genomic technologies. However, these clinicians seemed even less critical of commercial genomic risk assessment platforms than early DTC consumers that we interviewed for an earlier study [2], This is concerning because customers of DTC genomic profiling were cautioned to be watchful consumers, but critical reflection is assumed among those holding titles as clinical professionals and was not what we found among early adopting clinicians. Even if clinicians desire genomic knowledge, the DTP model of commercial marketing and test dissemination is unlikely to foster autonomous substantive expertise among clinicians.

Simply routing commercially-generated genomic risk information to patients through physicians will not meet the complex challenges posed by burgeoning markets in consumer and clinical genomics. To increase competent clinical judgment in genomics requires a more nuanced understanding of the dynamics of private clinical practice, how clinicians learn about new testing modalities, and the values that undergird clinical integration of genomic testing. Rather than emphasizing the need for expert interpretation of genomic test results, the mode of delivery itself requires further interrogation and regulatory oversight. Because commercial laboratories are the entities with the most “skin in the game” financially, these laboratories are not the most appropriate entities to hold and control genomic information and its interpretation. While SNP-based tests are likely to be replaced by NGS platforms [49], the dependence of clinicians on commercial genomics laboratories is likely to endure as increasingly complex genomic information is taken up by clinicians with less enthusiasm or interest in engaging genomics than the “early adopters” we interviewed. Regardless of the technological platform, further deliberation and guidelines from professional societies and federal oversight of commercial laboratory services is needed to demonstrate the analytic and clinical validity and clinical utility of genomic tests. The dependencies inherent in their interactions with clinicians pose risks to the integrity of clinical judgment and patient care. Left unattended, these risks will only increase as genomics is further integrated into clinical care.

## Supporting Information

Appendix S1 **Interview Guide: Clinical Practitioners of Personalized Genomic Medicine.**
(DOC)
