# Supplementary material for: Gatekeepers or Intermediaries? The Role of Clinicians in Commercial Genomic Testing
Source: PLoS One. 2014 Sep 26;9(9):e108484. doi: 10.1371/journal.pone.0108484 (PMC4178171; doi:10.1371/journal.pone.0108484)
Supplement: Appendix S1 — Interview Guide: Clinical Practitioners of Personalized Genomic Medicine. (DOC) [file pone.0108484.s001.doc]

**Appendix S1: Interview Guide: *Clinical Practitioners of Personalized Genomic Medicine***

**Background/Definitions**

1. Can you tell me about your background? Can you tell me about your clinical practice?

*Probe- If not known, get degrees, fields, and current position title*

2. How did you become a clinician partner of Navigenics/DNA Direct?

3. How did you become interested in personalized genomic medicine?

*Probe- What personal and/or professional experiences drew you into the field?*

4. What does the label “personalized genomic medicine” mean to you?

*Probe- In your view, what health care strategies or services does “personalized genomic medicine” encompass?*

**Daily Practice of PGM**

5. Is “personalized genomic medicine” an appropriate term to characterize your practice?

6. How do you incorporate genomic medicine into the other services that your provide?

*Probe* - How do your clinical services differ from traditional medical genetics?

7. Where did you gain expertise on personalized genomic medicine?

8. Who are the patients seeking genomic services? What do they expect when they come to see you?

*Probe- Where do they learn about the availability of genomic risk assessment?*

9. From the vantage point of clinical medicine, who are the key people who shape the work of clinicians in the field of personalized genomic medicine? How, exactly, do they exert their influence?

**Clinical Promises**

10. What do you think will be the impact of personalized genomic medicine on healthcare?

*FOR US- examples include increased focus on preventive medicine, proactive, participatory, personalized, lower health care costs, less disparity in health care etc*

11. How might PGM change how *medicine* is practiced on a daily basis? Can you give a concrete example?

*Probe- Will the changes resulting from PGM be incremental or rapid in pace, moderate or dramatic in effect*

12. Moving translational genomic research (TGR) into the clinic will likely yield some new types of professional roles and responsibilities. What new roles do you envision in order to implement PGM successfully?

13. Who do you think should be responsible for preparing professionals to take on new roles in the context of PGM?

14. How do you think new physicians should be trained in order to incorporate PGM into their knowledge base?

15. Would moving TGR into the clinic yield changes for the role of the patient?

16.How do you envision PGM becoming integrated into clinical medicine? Do you think that personalized genomic medicine is likely to become its own medical specialty?

*Probe -* *Why or why not?*

*Probe- How long might it take for advances of translational genomic research to be integrated (mainstreamed) into the clinic?*

17. What does the future of PGM look like? How do you expect your own practice to change over the next 10 years?

**Challenges Related to Clinical Application/Medicine**

18. What clinical challenges need to be addressed if personalized genomic medicine is to be realized?

*Probe-* What do you think will be the greatest challenges in getting *physicians* to embrace PGM in their daily practices?

19. How well or accurately do you think personalized genomic medicine is understood by your patients?

*Probe- What barriers exist relative to patient understanding of personalized genomic medicine? How might those barriers be overcome?*

*Probe – What do you see as your responsibilities in explaining genetics or genomics to patients?*

20. What challenges does the structure of the health care system pose to the delivery of personalized genomic medicine?

*For example professional boundaries, reimbursement policies, institutional priorities, personnel needs,*

21. How might clinical research be utilized to maximize the translation of research findings into clinical practice?

**Other Challenges**

22. What ethical, legal and social challenges need to be addressed if personalized genomic medicine is to be realized in the clinic?

*Probe: How does personalized genomic medicine pose special ethical issues in conducting research with (1) children, (2) persons with low income, and (3) minority populations?*

**Future of PGM**

23. Who do think has the most to gain from the realization of personalized genomic medicine? (e.g., scientists, politicians, clinicians, patient/disease groups)

24. Conversely, who might have the most to lose or might be the most vulnerable if it comes to fruition? (e.g., scientists, politicians, clinicians, patient/disease groups)

25. Where do you think the field will be in 10 years? )

*Probe- Are there additional comments that you’d like to share with us that were not covered in the interview?*

**GATHER DEMOGRAPHIC INFORMATION FOR NIH REPORTING**

Gender

Ethnicity

Race

Age
